# The influence of burnout on cardiovascular disease: a systematic review and meta-analysis

**DOI:** 10.3389/fpsyt.2024.1326745

**Published:** 2024-02-19

**Authors:** Awena John, Jean-Baptiste Bouillon-Minois, Reza Bagheri, Carole Pélissier, Barbara Charbotel, Pierre-Michel Llorca, Marek Zak, Ukadike C. Ugbolue, Julien S. Baker, Frederic Dutheil

**Affiliations:** ^1^ Université Clermont Auvergne, CHU Clermont-Ferrand, Occupational Medicine, Clermont-Ferrand, France; ^2^ Université Clermont Auvergne, CNRS, LaPSCo, Physiological and Psychosocial Stress, CHU Clermont-Ferrand, Emergency Medicine, Clermont-Ferrand, France; ^3^ Department of Exercise Physiology, University of Isfahan, Isfahan, Iran; ^4^ Université Jean Monnet Saint-Etienne, IFSTTAR, Université Lyon 1, UMRESTTE, CHU Saint-Etienne, Occupational Medicine, Saint-Etienne, France; ^5^ Université Lyon 1, UMRESTTE, CHU Lyon, Occupational Medicine, Lyon, France; ^6^ Université Clermont Auvergne, CNRS, Clermont Auvergne INP, Institut Pascal, CHU Clermont-Ferrand, Psychiatry, Clermont-Ferrand, France; ^7^ Institute of Health Sciences, Collegium Medicum, The Jan Kochanowski University of Kielce, Kielce, Poland; ^8^ School of Health and Life Sciences, Institute for Clinical Exercise & Health Science, University of the West of Scotland, South Lanarkshire, United Kingdom; ^9^ Centre for Health and Exercise Science Research, Hong Kong Baptist University, Hong Kong, Hong Kong SAR, China; ^10^ Université Clermont Auvergne, CNRS, LaPSCo, Physiological and Psychosocial Stress, CHU Cler-mont-Ferrand, Occupational Medicine, WittyFit, Clermont-Ferrand, France

**Keywords:** mental health, burnout, cardiovascular disease, methodology, statistics

## Abstract

**Background:**

Burnout is a public health problem with various health consequences, among which cardiovascular disease is the most investigated but still under debate. Our objective was to conduct a systematic review and meta-analysis on the influence of burnout on cardiovascular disease.

**Methods:**

Studies reporting risk (odds ratio, relative risk, and hazard ratio) of cardiovascular disease following burnout were searched in PubMed, PsycINFO, Cochrane, Embase, and ScienceDirect. We performed a random-effect meta-analysis stratified by type of cardiovascular disease and searched for putative influencing variables. We performed sensitivity analyses using the most adjusted models and crude risks.

**Results:**

We included 25 studies in the systematic review and 9 studies in the meta-analysis (4 cross-sectional, 4 cohort, and 1 case–control study) for a total of 26,916 participants. Burnout increased the risk of cardiovascular disease by 21% (OR = 1.21, 95% CI 1.03 to 1.39) using the most adjusted risks and by 27% (OR = 1.27, 95% CI 1.10 to 1.43) using crude risks. Using stratification by type of cardiovascular disease and the most adjusted risks, having experienced burnout significantly increased the risk of prehypertension by 85% (OR = 1.85, 95% CI 1.00 to 2.70) and cardiovascular disease-related hospitalization by 10% (OR = 1.10, 95% CI 1.02 to 1.18), whereas the risk increase for coronary heart disease (OR = 1.79, 95% CI 0.79 to 2.79) and myocardial infarction (OR = 1.78, 95% CI 0.85 to 2.71) was not significant. Results were also similar using crude odds ratio. The risk of cardiovascular disease after a burnout was not influenced by gender. Insufficient data precluded other meta-regressions.

**Conclusions:**

Burnout seems to increase the risk of cardiovascular disease, despite the few retrieved studies and a causality weakened by cross-sectional studies. However, numerous studies focused on the pathophysiology of cardiovascular risk linked to burnout, which may help to build a preventive strategy in the workplace.

## Introduction

Burnout is a public health problem (1). The term burnout was first used in 1969 by Harold B. Bradley (2). This term was taken up in 1974 by the psychoanalyst Herbert J. Freudenberger (3) and then in 1976 by the psychologist Christina Maslach (2). According to the World Health Organization, burnout is a syndrome combining a sense of exhaustion, cynicism or increased mental distance from work, and decreased work effectiveness, resulting from chronic stress at work that has not been successfully managed (4). Among the various health consequences of burnout, cardiovascular disease remains the most investigated and debated (5). Cardiovascular disease is the leading cause of death in the world, causing approximately 17.9 million deaths each year (6). However, cardiovascular disease promoted by burnout is not well known. Cardiovascular disease is a very diverse group of diseases, including diseases of the heart and blood vessels (7). Some studies (7, 8) investigated the pathophysiology of burnout, particularly the development of cardiovascular disease. Quantitative studies have limitations, in particular in their reporting of each type of cardiovascular disease. The calculation of risks seems heterogeneous between studies (9, 10). To date, no meta-analysis has specifically focused on the risk of cardiovascular disease following burnout exposure, putatively able to reconcile diverging literature. Individual risk factors for cardiovascular disease, such as age, gender, body mass index, smoking, physical activity, and lipid levels (11–13), were also not investigated specifically in the context of burnout.

Therefore, our objective is to perform a systematic review and meta-analysis on the influence of burnout on cardiovascular disease. A secondary objective is to compare the influence of burnout according to each cardiovascular disease and to study individual risk factors that influence the risk of cardiovascular disease due to burnout.

## Methods

### Literature search

**Figure 1:**
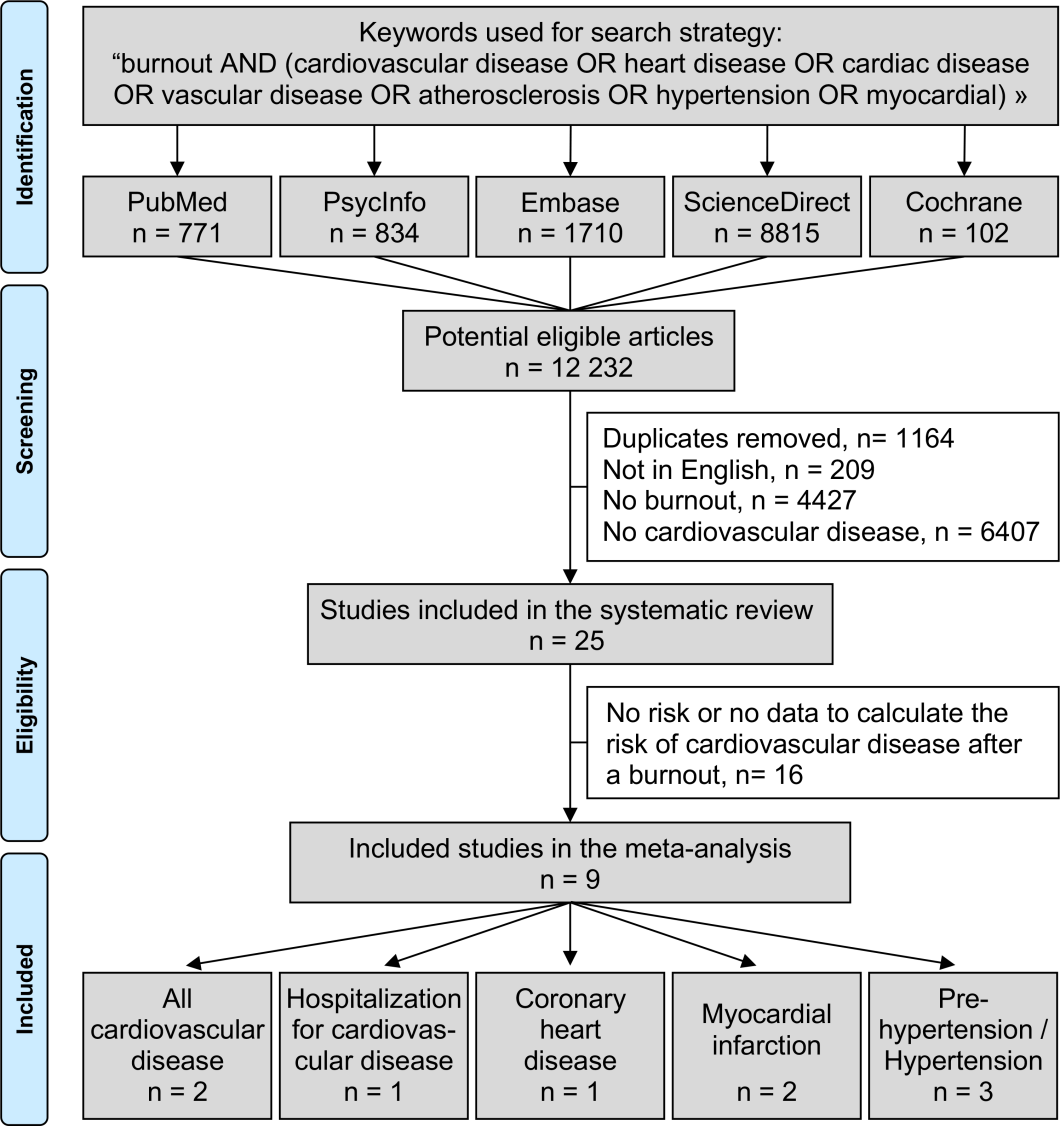
Search strategy.

The following PICO (population, investigated condition, i.e., exposure, comparisons, and outcome) question was formulated: Are workers exposed to burnout at higher risk of cardiovascular disease, compared to workers not exposed to burnout? We reviewed all studies reporting the risk of having cardiovascular disease in relation with burnout. The PubMed, Cochrane Library, Embase, ScienceDirect, and PsycINFO databases were searched in July 2022 with the following keywords: burnout AND (cardiovascular disease OR heart disease OR cardiac disease OR vascular disease OR atherosclerosis OR hypertension OR myocardial) (details for the search strategy used within each database are available in **Appendix 1**). We limited our search to articles in adult workers and to those written in English. To be included in the systematic review, studies needed to describe our primary outcome variable, i.e., the influence of burnout on cardiovascular disease. Articles describing either an odds ratio, a relative risk, or a hazard ratio, or giving data to calculate the risk of cardiovascular disease following a burnout, were included in the meta-analysis. We included any type or diagnosis of cardiovascular disease including high blood pressure and atherosclerosis, but not dyslipidemia as we considered it more related to metabolic disease. For high blood pressure, we considered all cutoffs defined in retrieved articles. We also considered outcomes related to cardiovascular disease such as hospitalization for cardiovascular disease. References from all publications meeting the inclusion criteria were also searched manually to identify potential additional studies that were not found during the electronic search. In addition, we performed ancestry searches to locate other potentially eligible primary studies from previous reviews. Two authors (Awena John and Jean-Baptiste Bouillon-Minois) conducted the literature searches, reviewed the abstracts, and, based on the selection criteria, decided the suitability of the articles for inclusion and extracted the data. When necessary, disagreements were solved with a third author (Frédéric Dutheil) (**Figure 1**). Then, all authors reviewed the eligible articles.

### Data extraction

The data collected included first author’s name, publication year, aims, outcomes of included articles, characteristics of studies (study design, periods of collection of data, and country), characteristics of the population (sample size, age, sex, and occupation), characteristics of burnout (scale used, method of calculation, and thresholds), characteristics of cardiovascular disease (type and criteria for diagnosis), and its associated risk (method of calculation, type of risk, and adjustment models).

### Quality of assessment

**Figure 2:**
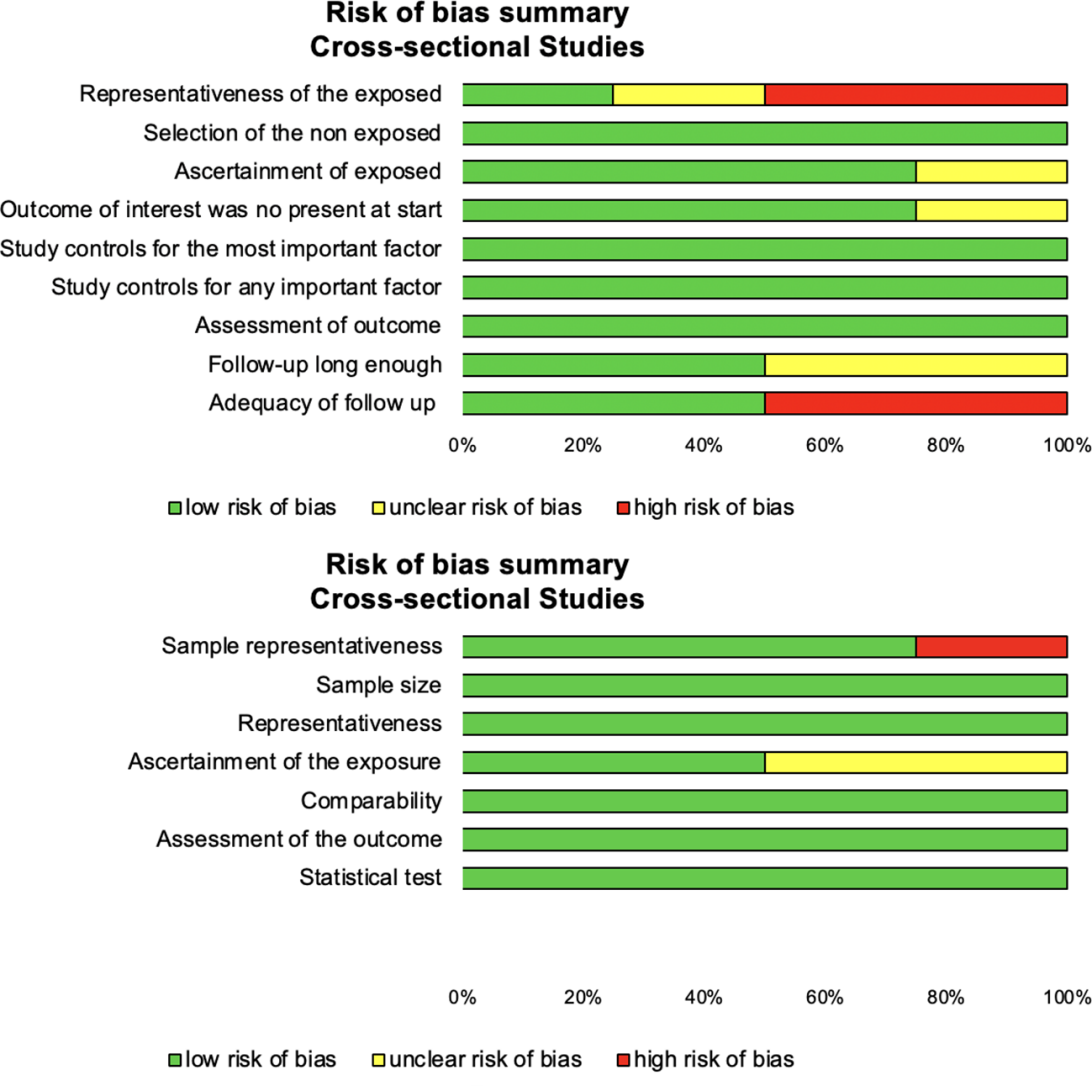
Methodological quality of included articles.

We used the Newcastle–Ottawa Scale (NOS) to check the quality of included articles (14) (**Figure 2**). The maximum score was 9 for cohort and 10 for cross-sectional studies. Additionally, we also used the Strengthening the Reporting of Observational Studies in Epidemiology (STROBE) for cohort and cross-sectional studies (15) (**Appendix 2**).

### Statistical considerations

**Figure 3:**
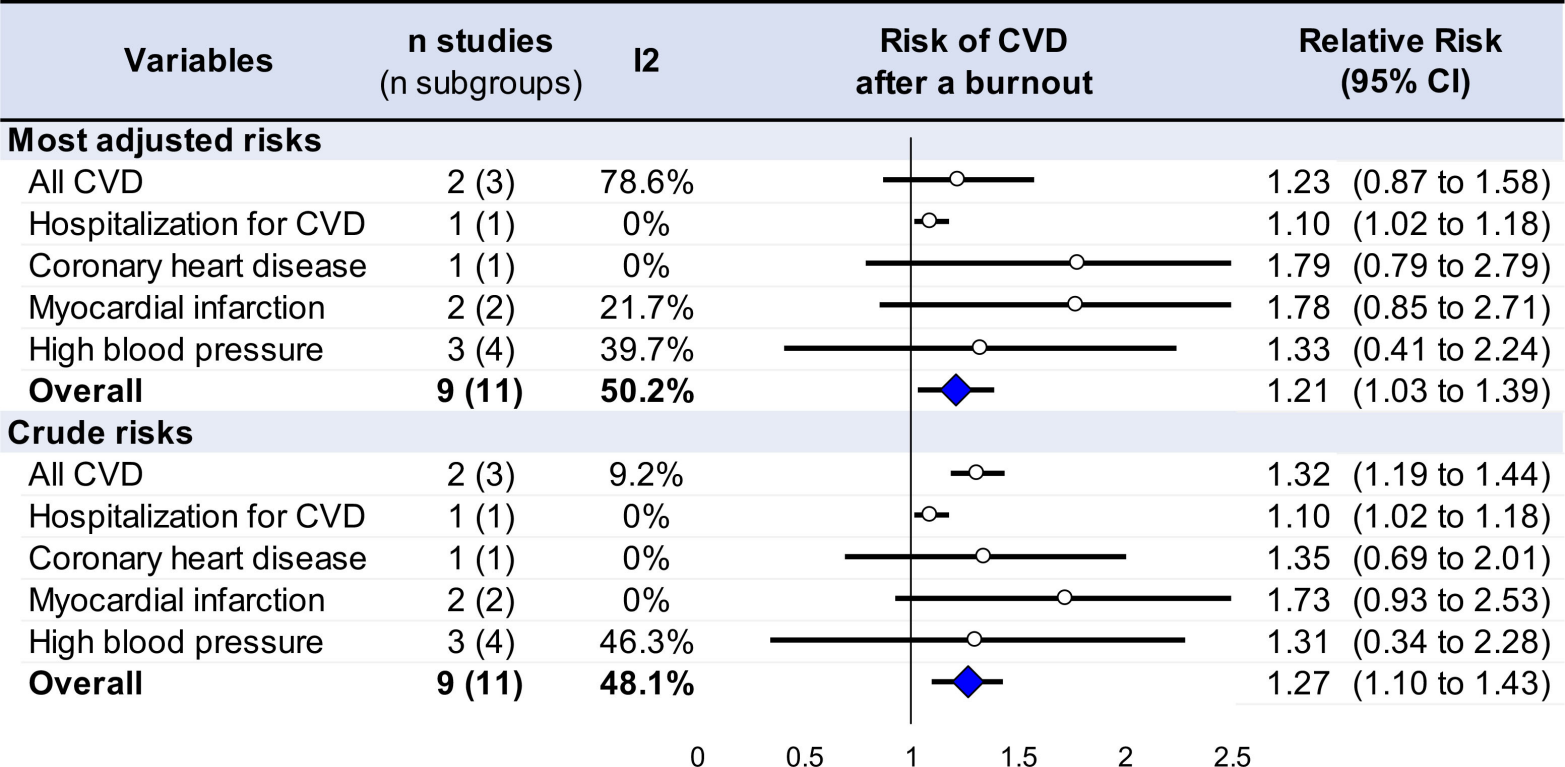
Summary of meta-analyses on the risk of cardiovascular disease after a burnout.

Statistical analysis was conducted using Stata software (v16, StataCorp, College Station, USA) (16–20). Extracted data were summarized for each study and reported as mean (standard deviation) and number (%) for continuous and categorical variables, respectively. We conducted random-effects meta-analysis (DerSimonian and Laird approach) (21) on the risk of cardiovascular disease following burnout using all odds ratio, relative risk, or hazard ratio (22–26). We stratified this meta-analysis on the type of cardiovascular disease (all cardiovascular disease, hospitalization for cardiovascular disease, coronary heart disease (CHD), myocardial infarction, and high blood pressure). We described our results by calculating the risk of cardiovascular disease after a burnout (21). Risks were centered at one if the risk of cardiovascular disease after a burnout did not differ from the risk of cardiovascular disease without having any burnout. Risk > 1 denoted an increased risk of cardiovascular disease, and hazard ratio < 1 reflected a decreased risk. We conducted a meta-analysis on the most adjusted models and on crude risks (sensitivity analyses) (**Figure 3**, **Appendix 3**). Statistical heterogeneity between studies was assessed using forest plots, confidence intervals, and *I*²: heterogeneity is considered low for *I*² <25%, modest for 25%–50%, and high for >50%. We also aimed to conduct sensitivity analysis by excluding studies not evenly distributed around the base of the metafunnel (**Appendix 4**). We also proposed meta-regressions to investigate putative factors influencing the risk of cardiovascular disease following burnout exposure such as age, sex, occupation, or type of cardiovascular disease. Results were expressed as regression coefficients and 95% confidence intervals. Type I -error was fixed at α = 0.05.

## Results

An initial search produced 12,232 possible articles. Removal of duplicates and use of selection criteria reduced the number of articles reporting the influence of burnout on cardiovascular disease to 25 articles in the systematic review (5, 8–10, 27–47) and 9 were included in the meta-analysis (9, 10, 29, 36, 37, 41–44) (**Figure 1**). The main characteristics of all studies are outlined in **Table 1** and studies included in the meta-analyses are further described below. All articles were written in English.

**Table 1:** Characteristics of included studies in the meta-analysis.

Study	Country	Period of data collection	Design	Population				Burnout		
				*n*	Sex, % men	Age, years mean ± SD	Occupational work	Measure	Risk	Adjustment
Appels 1991 (9)	Netherland	1979–1983	Cohort	3,210	100	Burned out 52.3 ± 8.4 Controls 51.3 ± 8.6	64% blue collar workers	Maastricht Questionnaire	RR	Age, cholesterol, BP, smoking
Azfar 2021 (29)	Kyrgyzstan	Not precise	Cross-sectional	694	43.5	Mean age for six ethnic groups, from 34.3 ± 14.3 to 48.5 ± 15.7		SMBQ	OR	Ethnicity, gender and BMI
Honkonen 2005 (36)	Finland	2000–2001	Cross-sectional	3,368	52.3	44.6± 8.6	30% blue collar 28% upper white collar 27% lower white collar 15% self-employed	MBI-GS	OR	Sociodemographic, health behavior and depressive symptoms
Kitaoka 2009 (10)	Japan	4–5 years	Cohort	383	100	37.8 ± 11.6	Middle managers	MBI-GS (Japanese Version)	OR	Age, alcohol, smoking, physical activity
Lin 2021 (37)	Taiwan	Not precise	Cross-sectional	242	26.4	50.4±?	Workers in elderly welfare facilities	Copenhagen Burnout Inventory (Chinese Version)	OR	Age, sex, education, type of work shifts, nurse assistant work category, personal burnout, BMI
Sokejima 1998 (41)	Japan	1990–1993	Case–control	526	100	Burned out 55.5 ± 8.6 Controls 54.4 ± 8.3	51% managers and officials 15% professional and technical	Burnout measure by Pines	OR	Age, occupational categories
Toker 2012 (42)	Israël	2003–2010	Cohort	8,838	63	CHD 53.5 ± 7.9 No CHD 44.9 ± 10.5		SMBM	HR	Age, sex, family history of CHD, smoking, subjective and objective workload, depression
Toppinen-Tanner 2009 (43)	Finland	Not precise	Cohort	7,897	76	>18	Forest industry employees	MBI-GS	HR	Age, sex, occupational status, physical environment, medication for hypertension and diabetes
Tsou 2020 (44)	Taiwan	2018–2019	Cross-sectional	1,758	4.2	Median 35.2	Nurses	Chinese Burnout Inventory	OR	Sex, sleep time, alcohol, physical activity, fruit and vegetable intake

MBI-GS, Maslach Burnout Inventory – General Survey; SMBM, Shirom Melamed Burnout Measure; SMBQ, Shirom Melamed Burnout Questionnaire; Burnout measure by Pines from (48).

### Quality of articles

Using the NOS criteria for cross-sectional studies demonstrated a low risk of bias, except for sample representativeness and ascertainment of exposure (**Figure 2**). NOS and STROBE evaluation for each included study are available in **Appendix 2**.

### Aims and outcomes of included articles

Six studies aimed to evaluate the association between burnout and the incidence or the prevalence of cardiovascular disease as a primary result (9, 10, 36, 37, 42, 43), while three studies evaluated this association as a secondary result (29, 41, 44). For one study (29), the main objective was to characterize different ethnic groups in Kyrgyzstan regarding cardiovascular disease and mental distress; for another study (41), it was to clarify the extent to which working hours affect the risk of acute myocardial infarction; and for the third study (44), it was to analyze the association between burnout and metabolic syndrome.

### Study designs

Four studies were cross-sectional (29, 36, 37, 44), four were cohort studies (9, 10, 42, 43), and one was a case–control study. Cohort studies had a follow-up from 3.6 (42) to 10 (43) years on average. Five studies were conducted in Asia [Kyrgyzstan (29), Japan (10, 41), and Taiwan (37, 44)], three in Europe [Netherlands (9) and Finland (36, 43)], and one in Israel (42).

### Recruitment of individuals

Recruitment procedures included individuals visiting polyclinics and healthcare centers (29, 41, 42), city employees (9), middle managers working on a manufacturing company (10), workers in public elderly welfare facilities (37), nurses working in a tertiary hospital (44), forest industry employees (43), and workers from the general population, i.e., any worker (36).

### Populations studied

Sample size ranged from 242 (37) to 8,838 (42). In total, 26,916 individuals were included in our meta-analysis.

Age was reported in all studies. Most of the studies (9, 10, 29, 36, 37, 41, 42) reported mean age. One study (44) reported age as a median, and one study (43) reported prevalence by age category.

Gender was reported in all studies. Overall, there were 43% of the sample were men (95% CI 20% to 66%), ranging from 26% (37) to 100% (9, 10, 41).

Other descriptive variables included body mass index (BMI) (10, 29, 36, 37, 41, 42), blood pressure (9, 10, 36, 37, 41–44), cholesterol (9, 10, 41, 42), occupational status (9, 36, 41, 43), work style (37, 42, 44), education level (29, 36, 37, 42), marital status (9, 29, 36), ethnicity (29), physical activity (10, 36, 42, 44), smoking habits (9, 10, 36, 41, 42, 44), and alcohol consumption (10, 36, 44), and two studies focused on family history of heart disease (41, 42). In addition, most studies (78%) conducted health examination to evaluate the risk of cardiovascular disease (9, 10, 36, 37, 41, 42, 44). The health examination included measurement of height (36, 41, 44), body weight (9, 36, 44), body circumference (10, 36), waist circumference (10, 44), electrocardiogram (ECG) (9, 36), spirometry (36), bioimpedance (36), heel bone density (36), blood test (10, 36), total cholesterol (9, 41), triglycerides, high-density lipoprotein cholesterol, and low-density lipoprotein cholesterol (42), fasting insulin and glucose tolerance (9, 42), and HbA1c and TSH (44) (**Table 1**).

### Measurements of burnout

All studies used self-administered questionnaires to assess burnout, but differed between studies except three studies (10, 36, 43) that used the Maslach Burnout Inventory – General Survey (MBI-GS) (49). The MBI-GS a 16-item questionnaire consisting of three subscales: exhaustion (five items), cynicism (five items), and lack of professional efficacy (six items), each item being scored on a seven-point scale ranging from 0 (“never”) to 6 (“daily”). One study used only four items for cynicism (10). Two studies (36, 43) calculated levels of burnout using a weighted sum: (0.4/5 × exhaustion + 0.3/5 × cynicism + 0.3/6 × lack of professional efficacy)/6 and considered burnout to be severe if the score is 3.50 to 6, mild if the score is 1.50 to 3.49, and no burnout is the score is 0 to 1.49. Despite not being written, the reference cited indicated that odds ratios for cardiovascular disease were calculated for the combination mild plus severe burnout. The third study (10) used previous data from their research team to divide each subscale into tertiles: burnout (yes/no) was considered for the combination of intense exhaustion (upper third) and either high cynicism (upper third) or low professional efficacy (lower third) or both. One study (29) used the Shirom Melamed Burnout Questionnaire (SMBQ) (38), which is an eight-item questionnaire, each item being scored on a seven-point scale from 1 (“almost never”) to 7 (“almost always”). Scores were summed and burnout was recorded as a dichotomous variable: high (uppermost quartile) and low (other quartiles). Another study (42) used the Shirom Melamed Burnout Measure (SMBM) (50), which is a 14-item questionnaire, also scored on a seven-point scale. Burnout was also recorded as a dichotomous variable: high (uppermost quintile) and low (other quintiles). One study (35) used the Chinese version of the Copenhagen Burnout Inventory (51), which is a 13-item questionnaire (items 1 to 6 for personal burnout and items 7 to 13 for work-related burnout), each item being scored on a five-point scale: 0 (“almost never”), 25 (“never”), 50 (“sometimes”), 75 (“often”), and 100 (“always”). Scores of items 7 to 13 were averaged, and work-related burnout was considered mild for a score <45, moderate for 50 to 69.9, and severe for ≥70. Odds ratios for cardiovascular disease were calculated for the combination moderate plus severe burnout (score >45). One study (44) used the Chinese occupational burnout inventory (52) that consists of three domains: psychological work demands, job control/personal accomplishment, and employment stability. After summing points for each domain, burnout (yes/no) was defined as high psychological work demands (upper tertile) plus low job control (lower tertile) plus low employment stability (lower tertile). The case–control study (41) used the “Burnout measure”, a 21-item questionnaire, developed by Pines (48), consisting of three subscales (physical, emotional, and mental exhaustion), with items being scored on a seven-point scale ranging from 1 (“never”) to 7 (“always”). Calculation of scores was not written but the reference cited (53) averaged scores of each item, and defined burnout as a dichotomous variable: burnout (average score >4) and no burnout (<4). A third category of “borderline” burnout was presented but not explained. We only considered odds ratio for burnout. The last study (9) added the question “Have you ever been burned out” (categorical answer: yes/no) to the Maastricht Questionnaire that addresses loss of energy, increased irritability, and demoralization, which was constructed to be an indicator of mental precursors to myocardial infarction (54). Prevalence of cardiovascular diseases by dimension of burnout (exhaustion, cynicism, and lack of professional efficacy) was reported in one study (44) and by severity per dimension in one study (36), and odds ratio per dimension of burnout was reported in one study (43).

### Measurements of cardiovascular disease

All cardiovascular diseases were reported in two studies (29, 36). Diagnosis of cardiovascular disease was assessed using the question “Do you have any cardiovascular disease diagnosed by a doctor?” in one study (29), and using the Symptom Interview that was carried out in the first part of the health examination in the second study (36).

Hospitalization for cardiovascular disease was assessed in one study (43). Information on hospital admissions data was retrieved from the National Hospital Discharge Register, which is a complete, reliable source of illnesses that includes all hospital admissions with their causes.

CHD was assessed in one study (42). CHDs were defined as a composite of acute myocardial infarction, ischemic heart disease, and angina pectoris. Participants had to complete a self-report of medically diagnosed CHD.

Myocardial infarction was assessed in two studies (9, 41). One study (41) included patients admitted for a first attack of acute myocardial infarction. The other study (9) followed a cohort of patients with angina pectoris and retrieved those who had a myocardial infarction during the follow-up (54). In both studies, the diagnosis criteria for myocardial infarction were based on typical chest pain, electrocardiogram, and enzyme levels (55).

High blood pressure was reported in three studies: prehypertension was defined as systolic and diastolic blood pressure between 120/80 and 139/89 mmHg (37); ≥130/85 mmHg in the metabolic syndrome (44); and hypertension was defined as ≥140/90 mmHg (10, 37).

### Meta-analysis on the risk of cardiovascular disease after burnout

Overall using the most adjusted risks, the overall risk of cardiovascular disease increased by 21% following burnout exposure (OR = 1.21, 95% CI 1.03 to 1.39). Using stratification by type of cardiovascular disease, having a burnout significantly increased the risk of cardiovascular disease-related hospitalization by 10% (1.10, 1.02 to 1.18) (43), and prehypertension by 85% (1.85, 1.00 to 2.70) (37, 44) (**Figure 3**, **Appendix 3**). Risks were not significant for CHD (1.79, 0.79 to 2.79) (42), myocardial infarction (1.78, 0.85 to 2.71) (9, 41), and hypertension (0.69, −0.07 to 1.45) (10, 37).

### Sensitivity analyses and meta-regressions

We repeated an aforementioned meta-analysis using crude odds ratio and found similar results, i.e., an overall risk of cardiovascular disease following burnout exposure (OR = 1.32, 95% CI 1.19 to 1.44), as well as the risk of cardiovascular disease-related hospitalization (1.10, 1.02 to 1.18) and prehypertension (1.85, 1.00 to 2.70) (**Figure 3**, **Appendix 3**). Metafunnels analyzing for potential publication bias are presented in **Appendix 4**; however, excluding studies outside of metafunnels was deemed impossible because of the limited number of included studies.

The risk of cardiovascular disease after burnout was not influenced by gender (coefficient −0.007% men, 95% CI −0.071 to 0.056). Insufficient data precluded other meta-regressions.

## Discussion

Despite the few numbers of studies and the fact that the causality is weakened by the cross-sectional design of some studies, the main finding was that burnout seems to increase the risk of cardiovascular disease. Insufficient data precluded further analyses of other risk factors. However, numerous studies focused on the pathophysiology of the cardiovascular risk linked to burnout.

### Influence of burnout on cardiovascular disease: a public health issue

By the 1940s, cardiovascular disease became the number one cause of mortality among Americans, accounting for one in two deaths (56). Prevention and treatment were so poorly understood that most Americans accepted early death from heart disease as unavoidable (57). The Framingham study conducted in 1948 highlighted the importance of prevention in the occurrence of cardiovascular disease in individuals at high risk (57). A key component of this strategy was the ability to identify those most likely to have a future cardiovascular event, so that preventive interventions could be targeted (57). However, occupational risk factors were not evaluated or even reported in those historical studies. In 2004, the Interheart case–control study was the first large-scale study that also investigated occupational characteristics as risk factors for cardiovascular events. The study found that stress at work was involved in the occurrence of cardiovascular events (58). If stress at work has been studied for a long time, burnout is a more recent concept (59). The possibility that burnout may be a risk factor for the development of cardiovascular disease has been suggested in the 1990s (38), and the first prospective study using a valid measure of burnout was done in 2012, associating burnout with an increased risk of CHD (42). Despite the few numbers of studies and the fact that a causal relation could be extracted only from longitudinal studies, our meta-analysis seems in favor of an increased risk of cardiovascular disease following burnout, probably between 20% and 30%. Cardiovascular disease and burnout have both consequences on mental and physical health, as well as on work organization and economy (22, 23, 31–33, 37–40), increasing the numbers of sick leave and absenteeism (5). Interestingly, burnout also increases presenteeism, i.e., when people come to work even when sick, leading to a loss of productivity (5). In a vicious cycle, workers in burnout may not reach the desirable performance at work, which may lead to emotional exhaustion (60, 61). In addition to organizational and economic consequences for the companies, absenteeism or presenteeism related to burnout may represent the beginning of a social decline involving job loss and even permanent exclusion from the labor market (5).

### Depending on individual risk factors

Age over 45 years old is a common risk factor for burnout and cardiovascular disease, while being a man is specifically a risk for cardiovascular disease (62), and being a woman specifically increases the risk of burnout (63). There are also risk factors related to occupation (44). Occupational factors that can both impact the risk of burnout and the risk of cardiovascular disease are heavy workload, long working hours, time pressure, low rewards, low autonomy (decision latitude), value conflict, and lack of clarity in goals (52). Those factors are considered common factors of stress. The more stressful the job, the higher the risk of burnout and cardiovascular disease (64). Preventing excessive work stress is a legal obligation in several countries, and promoting awareness of the link between stress and health among both employers and workers is an important component of workplace health promotion (65). Besides sociodemographic and occupational factors, lifestyle behaviors also play a role in the risk of burnout and cardiovascular disease (66). Smoking and alcohol negatively influence the incidence of both burnout and cardiovascular disease while physical activity reduces the risk of developing burnout or cardiovascular disease (42, 43). Interestingly, physical inactivity has been shown to be the only significant factor linked to stress, and it is commonly admitted that workplace health promotion should also encourage workers to exercise regularly (67). Specific ethnic characteristics also seem to influence the occurrence of burnout and cardiovascular disease, but the pathophysiology needs to be further explored (29). Besides putative genetic mechanisms related to ethnicity (68, 69), ethnic factors influencing the risk of burnout and cardiovascular disease could be better associated with common characteristics linked to family history, such as religious practices, geographical origins, or education (70–72). Indeed, despite the fact that, for example, having an Asian ethnicity has a lower risk of atherosclerosis (73), persistent ethnic and racial differences in all-cause and cardiovascular mortality are largely attributable to social determinants of health—i.e., poor social conditions are linked to mortality (74). At a state level, governments should act on systemic factors that shape health differences across racial and ethnic groups (74). Very interestingly, despite the fact that burnout seems ubiquitous, i.e., any worker could be in burnout, without evidence of a difference in burnout by ethnicity (75, 76), it has been shown in some articles that underrepresented minorities may have a lower prevalence of burnout (77, 78). A putative explanation could be because they form communities with stronger support groups, and also because the cultural diversity may be a protective factor from some working conditions (77, 78). To prevent burnout and cardiovascular disease, occupational health departments should consider relevant risk factors to identify high-risk groups for efficient preventive strategy (37).

### Burnout and cardiovascular disease pathophysiological similarities

Interestingly, the pathophysiology of burnout also shares some similarities with the pathophysiology of cardiovascular disease, through neuroendocrine and inflammatory responses, as well as metabolic processes (79). The dysregulation of the hypothalamic–pituitary–adrenal (HPA) axis has been demonstrated to play a major role in the pathophysiology of cardiovascular disease from historical studies (80–82). Interestingly, some studies suggest that burnout is also associated with dysregulation of the HPA axis (a key stress responsive endocrine system), proinflammatory cytokine levels, inflammation biomarkers, and higher allostatic load (8). Burnout is associated with functional disconnection between the amygdala and the anterior cingulate/medial prefrontal cortex (83). The HPA axis is the primary means through which humans mediate the response to stress (84). With prolonged chronic exposure to stress, the HPA axis negative feedback loop can be lost, rendering the mechanism dysfunctional (30). Repeated exposure to psychosocial stress results in exaggerated activation of both the HPA axis and the sympathetic nervous system (85). This maladaptive process has been implicated in the development of burnout (27) and in cardiovascular disease because it disrupts metabolic parameters such as lipids, hypertension, and type 2 diabetes (30). The hyperstimulation of the HPA axis because of chronic stress also increases cortisol secretion (44). High levels of cortisol further suppress the development of new neurons in the hippocampus. Then, without resolution, limbic brain structures begin to atrophy (86). Reduced activity of the parasympathetic nervous system increases the risk of CHD (87, 88). It has been suggested that parasympathetic nerve activity is decreased by weak stressors that do not increase sympathetic nerve activity (89). Even if the stressors encountered while working are weak, coronary risk could be increased by attenuated vagal tone (30, 90).

### Limitations

Our study has some limitations. Our meta-analysis inherits the limitations of each included study. First of all, the interpretation of causality may be limited. Cross-sectional studies (29, 36, 37, 44) showed associations between burnout and cardiovascular disease; however, prospective studies are more appropriate for investigating the possible consequences of burnout (5). In our meta-analysis, few studies were prospective (9, 10, 42, 43), and a causal relation could be extracted only from longitudinal studies. Moreover, they had a short follow-up that may have restricted the ability to observe the effects of burnout on cardiovascular disease. Then, only published articles were included in our meta-analysis; thus, our results were theoretically exposed to a publication bias (91). We also included only studies written in English; hence, results were theoretically exposed to a selection bias. There were also wide variability in study populations (30), with some studies not being representative of the general working population (29, 42, 43), especially because ethnic groups differed significantly in age, gender, and education (29). However, taken together, this wide variability population is in favor of the generalizability of our results. Our meta-analysis also had limitations in the measurement of both burnout and cardiovascular disease. Indeed, burnout scales differed between studies, rendering comparisons less precise, even if all studies had a control group with no burnout. Assessment of burnout has always been reported through self-administered questionnaires, which may have led to an over- or underestimation of burnout. However, assessing a mental state is obviously subjective (92–94). Some studies also assessed cardiovascular disease using self-reported questionnaire (29, 36, 42). Cardiovascular disease was very diverse and heterogenous, decreasing the level of proof of the overall results of our meta-analysis. Lastly, except for gender, there were not enough data to analyze the putative influencing factors of the effects of burnout on cardiovascular diseases. For example, no meta-regressions were made on severity and duration of burnout, as well as on occupational characteristics, because they were not reported.

### Perspective of improvement for future studies

Considering that a causal link between burnout and cardiovascular disease is weakened by aforementioned limitations (particularly the inclusion of cross-sectional studies and the short follow-up for cohort studies), our meta-analysis also showed the need for further prospective studies with a long-term follow-up, using reliable records of cardiovascular disease as well as basic information such as details surrounding burnout (intensity and duration), sociodemographic factors (age, gender, marital status, and education level), occupational characteristics (job, occupational sector, and number of hours of work per week seniority), and lifestyle behavior (smoking, alcohol, physical activity, and nutrition/body mass index). Adjuvant measures should be assessed with reliable facility’s equipment (37). A long period of follow-up is needed because cardiovascular disease may occur several years after a burnout exposure, but a long period of follow-up is also needed to study the time effect itself for burnout to promote cardiovascular disease. To our knowledge, no study assessed the time period between burnout and cardiovascular disease, and associated influencing factors of this delay. Further studies should also use identical methods to measure burnout and to diagnose cardiovascular disease. All cardiovascular diseases should be considered, to assess disease-specific relationships.

## Conclusion

Despite the few numbers of included studies and a causality weakened by the cross-sectional design of some studies, our meta-analysis seems to be in favor of an increased risk of cardiovascular disease following burnout, probably between 20% and 30%. However, numerous studies focused on the pathophysiology of cardiovascular risk linked to burnout. This better understanding may help to build a preventive and efficient strategy in the workplace. Future studies should be achieved through prospective studies with a long-term follow-up, which may also help to understand the chronology of development of cardiovascular disease following burnout.

## Data availability statement

The original contributions presented in the study are included in the article/**Supplementary Material**. Further inquiries can be directed to the corresponding author.

## Author contributions

AJ: Writing – original draft. J-BB-M: Writing – original draft. RB: Writing – review & editing. CP: Writing – review & editing. BC: Writing – review & editing. P-ML: Writing – review & editing. MZ: Writing – review & editing. UU: Writing – review & editing. JB: Writing – review & editing. FD: Writing – original draft.

## Supplementary material

Appendix 1 Details for the search strategy used within each database.

Appendix 2 Details for methodological quality of included articles (Methodological assessment of studies using STROBE criteria, Methodological assessment of studies using NOS criteria) and Checklists used for quality assessment of included studies (Newcastle-Ottawa Scale (NOS) for cross-sectional studies, Newcastle-Ottawa Scale (NOS) for cohort studies, Strengthening the Reporting of Observational studies in Epidemiology (STROBE) for cross-sectional and cohort studies)

Appendix 3 Meta-analysis using the most adjusted and crude risk of cardiovascular disease after a burnout.

Appendix 4 Metafunnels, based on data from the most adjusted and crude risk of cardiovascular disease after a burnout.Each dot represents a single study, with its corresponding risk (x axis) and its associated standard error of the risk (y-axis). Large high-powered studies are placed towards the top, and smaller low-powered studies towards the bottom. The plot should ideally resemble a pyramid or inverted funnel, with scatter due to sampling variation. Studies outside funnel plot are likely to present bias (94).

## References

[1] Friganović A Selič P Ilić B Sedić B . Stress and burnout syndrome and their associations with coping and job satisfaction in critical care nurses: a literature review. Psychiatr Danub mars (2019) 31(Suppl 1):21−31. 30946714

[2] Freudenberger HJ . Syndrome d’épuisement professionnel. 23:.

[3] Freudenberger HJ . Staff burn-out. J Soc Issues (1974) 30(1):159−65. doi: 10.1111/j.1540-4560.1974.tb00706.x

[4] Burn-out an « occupational phenomenon »: International Classification of Diseases (2022). Available at: https://www.who.int/news/item/28-05-2019-burn-out-an-occupational-phenomenon-international-classification-of-diseases.

[5] Salvagioni DAJ Melanda FN Mesas AE González AD Gabani FL de Andrade SM . Physical, psychological and occupational consequences of job burnout: A systematic review of prospective studies. PloS One [Internet] (2017) 12(10). doi: 10.1371/journal.pone.0185781 PMC5627926 28977041

[6] Saheera S Krishnamurthy P . Cardiovascular changes associated with hypertensive heart disease and aging. Cell Transplant (2020) 29:0963689720920830. doi: 10.1177/0963689720920830 32393064 PMC7586256

[7] Maladies cardiovasculaires (2022). Available at: https://www.who.int/fr/health-topics/cardiovascular-diseases.

[8] Melamed S Shirom A Toker S Berliner S Shapira I . Burnout and risk of cardiovascular disease: Evidence, possible causal paths, and promising research directions. Psychol Bull (2006) 132(3):327−53. doi: 10.1037/0033-2909.132.3.327 16719565

[9] Appels A Schouten E . Burnout as a risk factor for coronary heart disease. Behav Med Wash DC (1991) 17(2):53−9. doi: 10.1080/08964289.1991.9935158 1878609

[10] Kitaoka-Higashiguchi K Morikawa Y Miura K Sakurai M Ishizaki M Kido T . Burnout and risk factors for arteriosclerotic disease: follow-up study. J Occup Health (2009) 51(2):123−31. doi: 10.1539/joh.L8104 19212087

[11] Fuchs FD Whelton PK . HIGH BLOOD PRESSURE AND CARDIOVASCULAR DISEASE. Hypertens Dallas Tex 1979 (2020) 75(2):285−92. doi: 10.1161/HYPERTENSIONAHA.119.14240 PMC10243231 31865786

[12] Jokinen E . Obesity and cardiovascular disease. Minerva Pediatr (2015) 67(1):25−32. 25387321

[13] Lavie CJ Ozemek C Carbone S Katzmarzyk PT Blair SN . Sedentary behavior, exercise, and cardiovascular health. Circ Res (2019) 124(5):799−815. doi: 10.1161/CIRCRESAHA.118.312669 30817262

[14] Stang A . Critical evaluation of the Newcastle-Ottawa scale for the assessment of the quality of nonrandomized studies in meta-analyses. Eur J Epidemiol (2010) 25(9):603−5. doi: 10.1007/s10654-010-9491-z 20652370

[15] Vandenbroucke JP . Strengthening the reporting of observational studies in epidemiology (STROBE): explanation and elaboration. Ann Intern Med (2007) 147(8). doi: 10.7326/0003-4819-147-8-200710160-00010-w1 17938389

[16] Bouillon-Minois JB Croizier C Baker JS Pereira B Moustafa F Outrey J . Tranexamic acid in non-traumatic intracranial bleeding: a systematic review and meta-analysis. Sci Rep (2021) 11(1):15275. doi: 10.1038/s41598-021-94727-y 34315966 PMC8316462

[17] Bruet S Butin M Dutheil F . Systematic review of high-flow nasal cannula versus continuous positive airway pressure for primary support in preterm infants. Arch Dis Child Fetal Neonatal Ed (2022) 107(1):56−9. doi: 10.1136/archdischild-2020-321094 34016651

[18] Dutheil F Aubert C Pereira B Dambrun M Moustafa F Mermillod M . Suicide among physicians and health-care workers: A systematic review and meta-analysis. PloS One (2019) 14(12):e0226361. 31830138 10.1371/journal.pone.0226361 PMC6907772

[19] Dutheil F Baker JS Mermillod M De Cesare M Vidal A Moustafa F . Shift work, and particularly permanent night shifts, promote dyslipidaemia: A systematic review and meta-analysis. Atherosclerosis (2020) 313:156−69. doi: 10.1016/j.atherosclerosis.2020.08.015 33069952

[20] Dutheil F Comptour A Mermillod M Pereira B Clinchamps M Charkhabi M . Letter to the Editor: Comment on « Maternal exposure to air pollution and risk of autism in children: A systematic review and meta-analysis ». Environ pollut Barking Essex 1987 (2020) 264:114724. doi: 10.1016/j.envpol.2020.114724 32559872

[21] DerSimonian R Laird N . Meta-analysis in clinical trials. Control Clin Trials (1986) 7(3):177−88. doi: 10.1016/0197-2456(86)90046-2 3802833

[22] Dutheil F Comptour A Morlon R Mermillod M Pereira B Baker JS . Autism spectrum disorder and air pollution: A systematic review and meta-analysis. Environ pollut Barking Essex 1987 (2021) 278:116856. doi: 10.1016/j.envpol.2021.116856 33714060

[23] Dutheil F Pélangeon S Duclos M Vorilhon P Mermillod M Baker JS . Protective effect on mortality of active commuting to work: A systematic review and meta-analysis. Sports Med Auckl NZ (2020) 50(12):2237−50. doi: 10.1007/s40279-020-01354-0 33034873

[24] Dutheil F Zaragoza-Civale L Pereira B Mermillod M Baker JS Schmidt J . Prostate cancer and asbestos: A systematic review and meta-analysis. Perm J (2020) 24. doi: 10.7812/TPP/19.086 PMC7039423 32097115

[25] Lamat H Sauvant-Rochat MP Tauveron I Bagheri R Ugbolue UC Maqdasi S . Metabolic syndrome and pesticides: A systematic review and meta-analysis. Environ pollut Barking Essex 1987 (2022) 305:119288. doi: 10.1016/j.envpol.2022.119288 35439599

[26] Mathieu S Naughton G Descatha A Soubrier M Dutheil F . Dupuytren’s Disease and exposure to vibration: Systematic review and Meta-analysis. Joint Bone Spine (2020) 87(3):203−7. doi: 10.1016/j.jbspin.2020.02.001 32061740

[27] Romito BT Okoro EN Ringqvist JRB Goff KL . Burnout and wellness: the anesthesiologist’s perspective. Am J Lifestyle Med (2021) 15(2):118−25. doi: 10.1177/1559827620911645 33786030 PMC7958220

[28] Alameri F Aldaheri N Almesmari S Basaloum M Albeshr NA Simsekler MCE . Burnout and cardiovascular risk in healthcare professionals during the COVID-19 pandemic. Front Psychiatry (2022) 13:867233. doi: 10.3389/fpsyt.2022.867233 35444572 PMC9014179

[29] Azfar HS Dzhusupov KO Orru H Nordin S Nordin M Orru K . Cardiovascular disease and mental distress among ethnic groups in Kyrgyzstan. Front Public Health (2021) 9:489092. doi: 10.3389/fpubh.2021.489092 34017812 PMC8129164

[30] Bayes A Tavella G Parker G . The biology of burnout: Causes and consequences. World J Biol Psychiatry (2021) 22(9):686−98. doi: 10.1080/15622975.2021.1907713 33783308

[31] Chang BP Gallos G Wasson L Edmondson D . The unique environmental influences of acute care settings on patient and physician well-being: A call to action. J Emerg Med (2018) 54(1):e19−21. doi: 10.1016/j.jemermed.2017.08.092 29329636 PMC6533904

[32] Clemow LP Pickering TG Davidson KW Schwartz JE Williams VP Shaffer JA . Stress management in the workplace for employees with hypertension: A randomized controlled trial. Transl Behav Med (2018) 8(5):761−70. doi: 10.1093/tbm/iby018 30202927 PMC6128963

[33] Denat Y Gokce S Gungor H Zencir C Akgullu C . Relationship of anxiety and burnout with extrasystoles in critical care nurses in Turkey. Pak J Med Sci (2016) 32(1):196−200. 27022374 10.12669/pjms.321.8407 PMC4795867

[34] Hallman T Burell G Setterlind S Odén A Lisspers J . Psychosocial risk factors for coronary heart disease, their importance compared with other risk factors and gender differences in sensitivity. J Cardiovasc Risk (2001) 8(1):39−49. doi: 10.1177/174182670100800106 11234725

[35] Hallman T Thomsson H Burell G Lisspers J Setterlind S . Stress, burnout and coping: differences between women with coronary heart disease and healthy matched women. J Health Psychol (2003) 8(4):433−45. doi: 10.1177/13591053030084003 19127710

[36] Honkonen T Ahola K Pertovaara M Isometsä E Kalimo R Nykyri E . The association between burnout and physical illness in the general population–results from the Finnish Health 2000 Study. J Psychosom Res (2006) 61(1):59−66. doi: 10.1016/j.jpsychores.2005.10.002 16813846

[37] Lin PY Chang CC Tung CY Chu WH Tong FG . Risk factors of prehypertension and hypertension among workers at public elderly welfare facilities in Taiwan: A cross-sectional survey. Med (Baltimore) (2021) 100(8):e24885. doi: 10.1097/MD.0000000000024885 PMC7909213 33663118

[38] Melamed S Kushnir T Shirom A . Burnout and risk factors for cardiovascular diseases. Behav Med (1992) 18(2):53−60. doi: 10.1080/08964289.1992.9935172 1392214

[39] Prosdócimo ACG Lucina LB Marcia O Jobs PMJ Schio NA Baldanzi FF . Prevalence of Burnout Syndrome in patients admitted with acute coronary syndrome. Arq Bras Cardiol (2015) 104(3):218−25. 25517388 10.5935/abc.20140196 PMC4386850

[40] Smaardijk VR Mommersteeg PMC Kop WJ Adlam D Maas AHEM . Psychological and clinical characteristics of female patients with spontaneous coronary artery dissection. J Neth Soc Cardiol Neth Heart Found (2020) 28(9):485−91. doi: 10.1007/s12471-020-01437-7 PMC7431500 32500434

[41] Sokejima S Kagamimori S . Working hours as a risk factor for acute myocardial infarction in Japan: case-control study. BMJ (1998) 317(7161):775−80. doi: 10.1136/bmj.317.7161.775 9740562 PMC28666

[42] Toker S Melamed S Berliner S Zeltser D Shapira I . Burnout and risk of coronary heart disease: a prospective study of 8838 employees. Psychosom Med (2012) 74(8):840−7. doi: 10.1097/PSY.0b013e31826c3174 23006431

[43] Toppinen-Tanner S Ahola K Koskinen A Väänänen A . Burnout predicts hospitalization for mental and cardiovascular disorders: 10-year prospective results from industrial sector. Stress Health J Int Soc Investig Stress (2009) 25(4):287−96. doi: 10.1002/smi.1282

[44] Tsou MT Pai TP Chiang TM Huang WH Lin HM Lee SC . Burnout and metabolic syndrome among different departments of medical center nurses in Taiwan-Cross-sectional study and biomarker research. J Occup Health janv (2021) 63(1):e12188. doi: 10.1002/1348-9585.12188 PMC7815683 33469969

[45] von Känel R Princip M Holzgang SA Fuchs WJ van Nuffel M Pazhenkottil AP . Relationship between job burnout and somatic diseases: a network analysis. Sci Rep (2020) 10(1):18438. 33116176 10.1038/s41598-020-75611-7 PMC7595180

[46] Zhang M Loerbroks A Li J . Job burnout predicts decline of health-related quality of life among employees with cardiovascular disease: A one-year follow-up study in female nurses. Gen Hosp Psychiatry (2018) 50:51−3. doi: 10.1016/j.genhosppsych.2017.10.004 29054016

[47] Zhang M Shi Y Yang Y Liu L Xiao J Guo T . Burnout is associated with poor recovery of physical performance and low quality of life in patients after their first episode of acute coronary syndrome: A hospital-based prospective cohort study. Int J Cardiol (2017) 227:503−7. doi: 10.1016/j.ijcard.2016.10.114 27836301

[48] Malach-Pines A . The burnout measure, short version. Int J Stress Manage (2005) 12(1):78−88. doi: 10.1037/1072-5245.12.1.78

[49] Schaufeli WB Leiter MP Maslach C Jackson SE . The MBI-General Survey. In: Maslach C Jackson SE Leiter MP , editors. Maslach Burnout Inventory Manual, 3rd ed. Palo Alto (Calif)7 Consulting Psychologist Press (1996). p. 19–26.

[50] Sassi N Neveu JP . Traduction et validation d’une nouvelle mesure d’épuisement professionnel: Le shirom-melamed burnout measure. [Translation and validation of a new measurement of professional exhaustion: The Shirom-Melamed Burnout Measure.]. Can J Behav Sci Rev Can Sci Comport (2010) 42(3):177−84.

[51] Yeh WY Cheng Y Chen CJ Hu PY Kristensen TS . Psychometric properties of the Chinese version of Copenhagen burnout inventory among employees in two companies in Taiwan. Int J Behav Med (2007) 14(3):126−33. doi: 10.1007/BF03000183 18062055

[52] Yeh WY Cheng Y Chen MJ Chiu AWH . Development and validation of an occupational burnout inventory. Taiwan J Public Health (2008) 27:349−64.

[53] Imai H . Burnout and work environments of public health nurses involved in mental health care. Occup Environ Med (2004) 61(9):764−8. doi: 10.1136/oem.2003.009134 15317917 PMC1763668

[54] Appels A Höppener P Mulder P . A questionnaire to assess premonitory symptoms of myocardial infarction. Int J Cardiol (1987) 17(1):15−24. 3666994 10.1016/0167-5273(87)90029-5

[55] Appels A Mulder P . Excess fatigue as a precursor of myocardial infarction. Eur Heart J (1988) 9(7). 10.1093/eurheartj/9.7.758 3169045

[56] Kannel WB . Contribution of the framingham study to preventive cardiology. J Am Coll Cardiol (1990) 15(1):206−11. 2136875 10.1016/0735-1097(90)90203-2

[57] Mahmood SS Levy D Vasan RS Wang TJ . The Framingham Heart Study and the epidemiology of cardiovascular disease: a historical perspective. Lancet Lond Engl (2014) 383(9921):999−1008. 10.1016/S0140-6736(13)61752-3 PMC4159698 24084292

[58] Yusuf S Hawken S Ounpuu S Dans T Avezum A Lanas F . Effect of potentially modifiable risk factors associated with myocardial infarction in 52 countries (the INTERHEART study): case-control study. Lancet Lond Engl (2004) 364(9438):937−52. 10.1016/S0140-6736(04)17018-9 15364185

[59] Maslach C Leiter MP . Understanding the burnout experience: recent research and its implications for psychiatry. World Psychiatry (2016) 15(2):103−11. doi: 10.1002/wps.20311 27265691 PMC4911781

[60] Aronsson G Gustafsson K . Sickness presenteeism: prevalence, attendance-pressure factors, and an outline of a model for research. J Occup Environ Med (2005) 47(9):958−66. doi: 10.1097/01.jom.0000177219.75677.17 16155481

[61] Wright TA Cropanzano R . Emotional exhaustion as a predictor of job performance and voluntary turnover. J Appl Psychol (1998) 83(3):486−93. doi: 10.1037/0021-9010.83.3.486 9648526

[62] Rodgers JL Jones J Bolleddu SI Vanthenapalli S Rodgers LE Shah K . Cardiovascular risks associated with gender and aging. J Cardiovasc Dev Dis (2019) 6(2):19. doi: 10.3390/jcdd6020019 31035613 PMC6616540

[63] Norlund S Reuterwall C Höög J Lindahl B Janlert U Birgander LS . Burnout, working conditions and gender - results from the northern Sweden MONICA Study. BMC Public Health (2010) 10:326. doi: 10.1186/1471-2458-10-326 20534136 PMC2896942

[64] Mirmohammadi SJ Taheri M Mehrparvar AH Heydari M Saadati Kanafi A Mostaghaci M . Occupational stress and cardiovascular risk factors in high-ranking government officials and office workers. Iran Red Crescent Med J (2014) 16(8):e11747. doi: 10.5812/ircmj.11747 25389469 PMC4221995

[65] Kivimäki M Kawachi I . Work stress as a risk factor for cardiovascular disease. Curr Cardiol Rep (2015) 17(9):74. 26238744 10.1007/s11886-015-0630-8 PMC4523692

[66] Lecca LI Campagna M Portoghese I Galletta M Mucci N Meloni M . Work related stress, well-being and cardiovascular risk among flight logistic workers: an observational study. Int J Environ Res Public Health (2018) 15(9):1952. doi: 10.3390/ijerph15091952 30205457 PMC6164722

[67] Chou LP Tsai CC Li CY Hu SC . Prevalence of cardiovascular health and its relationship with job strain: a cross-sectional study in Taiwanese medical employees. BMJ Open (2016) 6(4):e010467. doi: 10.1136/bmjopen-2015-010467 PMC4823424 27044581

[68] Khan N Palepu A Dodek P Salmon A Leitch H Ruzycki S . Cross-sectional survey on physician burnout during the COVID-19 pandemic in Vancouver, Canada: the role of gender, ethnicity and sexual orientation. BMJ Open (2021) 11(5):e050380. doi: 10.1136/bmjopen-2021-050380 PMC8111871 33972345

[69] Garcia LC Shanafelt TD West CP Sinsky CA Trockel MT Nedelec L . Burnout, depression, career satisfaction, and work-life integration by physician race/ethnicity. JAMA Netw Open (2020) 3(8):e2012762. doi: 10.1001/jamanetworkopen.2020.12762 32766802 PMC7414389

[70] Schultz WM Kelli HM Lisko JC Varghese T Shen J Sandesara P . Socioeconomic status and cardiovascular outcomes: challenges and interventions. Circulation (2018) 137(20):2166−78. doi: 10.1161/CIRCULATIONAHA.117.029652 29760227 PMC5958918

[71] Di Chiara T Scaglione A Corrao S Argano C Pinto A Scaglione R . Education and hypertension: impact on global cardiovascular risk. Acta Cardiol (2017) 72(5):507−13. doi: 10.1080/00015385.2017.1297626 28657499

[72] Rodrigues H Cobucci R Oliveira A Cabral JV Medeiros L Gurgel K . Burnout syndrome among medical residents: A systematic review and meta-analysis. PloS One (2018) 13(11):e0206840. doi: 10.1371/journal.pone.0206840 30418984 PMC6231624

[73] Mitchell C Korcarz CE Gepner AD Kaufman JD Post W Tracy R . Ultrasound carotid plaque features, cardiovascular disease risk factors and events: the multi-ethnic study of atherosclerosis. Atherosclerosis (2018) 276:195−202. 29970256 10.1016/j.atherosclerosis.2018.06.005 PMC7436944

[74] Post WS Watson KE Hansen S Folsom AR Szklo M Shea S . Racial/ethnic differences in all-cause and cardiovascular disease mortality: the multi-ethnic study of atherosclerosis (MESA). Circulation (2022) 146(3):229−39. 35861763 10.1161/CIRCULATIONAHA.122.059174 PMC9937428

[75] Lawrence JA Davis BA Corbette T Hill EV Williams DR Reede JY . Racial/ethnic differences in burnout: a systematic review. J Racial Ethn Health Disparities (2022) 9(1):257−69. doi: 10.1007/s40615-020-00950-0 33428158 PMC7799165

[76] Whitehead IO Moffatt S Jagger C Hanratty B . A national study of burnout and spiritual health in UK general practitioners during the COVID-19 pandemic. PloS One (2022) 17(11):e0276739. doi: 10.1371/journal.pone.0276739 36322555 PMC9629610

[77] Eltorki Y Abdallah O Riaz S Mahmoud S Saad M Ez-Eldeen N . Burnout among pharmacy professionals in Qatar: A cross-sectional study. PloS One (2022) 17(5):e0267438. doi: 10.1371/journal.pone.0267438 35511925 PMC9071121

[78] Douglas M Coman E Eden AR Abiola S Grumbach K . Lower likelihood of burnout among family physicians from underrepresented racial-ethnic groups. Ann Fam Med (2021) 19(4):342−50. doi: 10.1370/afm.2696 34264839 PMC8282293

[79] Woda A Picard P Dutheil F . Dysfunctional stress responses in chronic pain. Psychoneuroendocrinology (2016) 71:127−35. doi: 10.1016/j.psyneuen.2016.05.017 27262345

[80] Rosmond R Björntorp P . The hypothalamic–pituitary–adrenal axis activity as a predictor of cardiovascular disease, type 2 diabetes and stroke. J Intern Med (2000) 247(2):188−97. doi: 10.1046/j.1365-2796.2000.00603.x 10692081

[81] Gardner MP Lightman S Sayer AA Cooper C Cooper R Deeg D . Dysregulation of the hypothalamic pituitary adrenal (HPA) axis and physical performance at older ages: An individual participant meta-analysis. Psychoneuroendocrinology (2013) 38(1):40−9. doi: 10.1016/j.psyneuen.2012.04.016 22658392 PMC3533133

[82] Kumari M Shipley M Stafford M Kivimaki M . Association of diurnal patterns in salivary cortisol with all-cause and cardiovascular mortality: findings from the whitehall II study. J Clin Endocrinol Metab (2011) 96(5):1478−85. doi: 10.1210/jc.2010-2137 21346074 PMC3085201

[83] Jovanovic H Perski A Berglund H Savic I . Chronic stress is linked to 5-HT(1A) receptor changes and functional disintegration of the limbic networks. NeuroImage (2011) 55(3):1178−88. 21211567 10.1016/j.neuroimage.2010.12.060

[84] Smith SM Vale WW . The role of the hypothalamic-pituitary-adrenal axis in neuroendocrine responses to stress. Dialogues Clin Neurosci (2006) 8(4):383−95. doi: 10.31887/DCNS.2006.8.4/ssmith 17290797 PMC3181830

[85] Orosz A Federspiel A Haisch S Seeher C Dierks T Cattapan K . A biological perspective on differences and similarities between burnout and depression. Neurosci Biobehav Rev (2017) 73:112−22. doi: 10.1016/j.neubiorev.2016.12.005 27993607

[86] Pretty J Rogerson M Barton J . Green mind theory: how brain-body-behaviour links into natural and social environments for healthy habits. Int J Environ Res Public Health (2017) 14(7):E706. doi: 10.3390/ijerph14070706 PMC5551144 28665327

[87] Hayano J Sakakibara Y Yamada M Ohte N Fujinami T Yokoyama K . Decreased magnitude of heart rate spectral components in coronary artery disease. Its relation to angiographic severity. Circulation (1990) 81(4):1217−24. 2317904 10.1161/01.cir.81.4.1217

[88] Hayano J Yamada A Mukai S Sakakibara Y Yamada M Ohte N . Severity of coronary atherosclerosis correlates with the respiratory component of heart rate variability. Am Heart J (1991) 121(4 Pt 1):1070−9. doi: 10.1016/0002-8703(91)90664-4 2008828

[89] Mukai S Hayano J . Heart rate and blood pressure variabilities during graded head-up tilt. J Appl Physiol Bethesda Md 1985 (1995) 78(1):212−6. 10.1152/jappl.1995.78.1.212 7713814

[90] Shi Y Jiang R Zhu C Zhang M Cai H Hu Z . High job burnout predicts low heart rate variability in the working population after a first episode of acute coronary syndrome. Int J Environ Res Public Health (2021) 18(7). 10.3390/ijerph18073431 PMC8037205 33810217

[91] Sterne JAC Egger M Smith GD . Investigating and dealing with publication and other biases in meta-analysis. BMJ (2001) 323(7304):101−5. doi: 10.1136/bmj.323.7304.101 11451790 PMC1120714

[92] Clinchamps M Auclair C Prunet D Pfabigan D Lesage FX Baker JS . Burnout among hospital non-healthcare staff: influence of job demand-control-support, and effort-reward imbalance. J Occup Environ Med (2021) 63(1):e13−20. 33149005 10.1097/JOM.0000000000002072

[93] Dutheil F Parreira LM Eismann J Lesage FX Balayssac D Lambert C . Burnout in french general practitioners: A nationwide prospective study. Int J Environ Res Public Health (2021) 18(22):12044. 34831796 10.3390/ijerph182212044 PMC8624683

[94] Sérole C Auclair C Prunet D Charkhabi M Lesage FX Baker JS . The forgotten health-care occupations at risk of burnout-A burnout, job demand-control-support, and effort-reward imbalance survey. J Occup Environ Med (2021) 63(7):e416−25. 34184659 10.1097/JOM.0000000000002235

