# Supplementary material for: The influence of burnout on cardiovascular disease: a systematic review and meta-analysis
Source: Front Psychiatry. 2024 Feb 19;15:1326745. doi: 10.3389/fpsyt.2024.1326745 (PMC10909938; doi:10.3389/fpsyt.2024.1326745)

Supplement

Appendix 1. Details for the search strategy used within each database.

Appendix 2. Details for methodological quality of included articles (Methodological assessment of studies using STROBE criteria, Methodological assessment of studies using NOS criteria) and Checklists used for quality assessment of included studies (Newcastle-Ottawa Scale (NOS) for cross-sectional studies, Newcastle-Ottawa Scale (NOS) for cohort studies, Strengthening the Reporting of Observational studies in Epidemiology (STROBE) for cross-sectional and cohort studies)

Appendix 3. Meta-analysis using the most adjusted and crude risk of cardiovascular disease after a burnout.

Appendix 4. Metafunnels, based on data from the most adjusted and crude risk of cardiovascular disease after a burnout.

Each dot represents a single study, with its corresponding risk (x axis) and its associated standard error of the risk (y-axis). Large high-powered studies are placed towards the top, and smaller low-powered studies towards the bottom. The plot should ideally resemble a pyramid or inverted funnel, with scatter due to sampling variation. Studies outside funnel plot are likely to present bias (94).

Electronic Supplementary Material

Appendix 1. Details for the search strategy used within each database

Pubmed

"burnout, psychological" OR "burnout, professional" OR "burnout" OR "caregiver exhaustion" OR "burn-out")

AND

“heart” OR “heart disease” OR “heart diseases” OR "cardiac” OR “cardiac disease” OR “cardiac diseases” OR “cardiovascular disease" OR ("cardiovascular" AND "diseases") OR "cardiovascular diseases" OR ("cardiovascular" AND "disease") OR “vascular” OR “vascular disease” OR “vascular diseases” OR “atherosclerosis” OR “cholesterol” OR “hypertension” OR “myocardial”

Embase

"burnout"

AND

“heart” OR "cardiac” OR "cardiovascular disease” OR “vascular” OR “atherosclerosis” OR “cholesterol” OR “hypertension” OR “myocardial”

Cochrane

"burnout"

AND

“heart” OR "cardiac” OR "cardiovascular disease" OR “vascular” OR “atherosclerosis” OR “cholesterol” OR “hypertension” OR “myocardial”

ScienceDirect

burnout

AND

“heart” OR "cardiac” OR "cardiovascular disease" OR “vascular” OR “atherosclerosis” OR “cholesterol” OR “hypertension” OR “myocardial”

Psycinfo

burnout

AND

“heart” OR "cardiac” OR "cardiovascular disease" OR “vascular” OR “atherosclerosis” OR “cholesterol” OR “hypertension” OR “myocardial”

Appendix 2. Assessment of quality of studies using STROBE criteria, NOS criteria, and checklists used

Methodological assessment of studies using NOS criteria

cross-sectional studies

Study	Sample representativeness (Selection bias)	Sample size (Selection bias)	Representativeness (Selection bias)	Ascertainment of the exposure (Selection bias)	Comparability (Comparability bias)	Assessment of the outcome (Outcome bias)	Statistical test (Outcome bias)
Azfar, 2021	+	+	+	+	+	+	+
Honkonen, 2005	+	+	+	+	+	+	+
Lin, 2021	+	+	+	?	+	+	+
Tsou, 2020	-	+	+	?	+	+	+

cohort studies

Study	Representativeness of the exposed (Selection bias)	Selection of the non-exposed (Selection bias)	Ascertainment of the exposure (Selection bias)	Outcome not present at start of study (Selection bias)	Comparability (Comparability bias)	Assessment of the outcome (Outcome bias)	Follow-up long (Outcome bias)	Adequacy of follow-up (Outcome bias)
Appels, 1991	-	+	+	?	+	+	?	-
Kitaoka, 2009	?	+	?	+	+	+	?	+
Toker, 2012	+	+	+	+	+	+	+	+
Toppinen, 2009	-	+	+	+	+	+	+	-

Methodological assessment of studies using STROBE criteria

STROBE					
	Total score	Abstract Introduction	Methods	Results	Discussion
Appels, 1991	64	100	62	40	100
Azfar, 2021	51	100	62	10	75
Honkonen, 2005	67	100	69	40	100
Kitaoka, 2009	54	100	58	40	75
Lin, 2021	58	100	54	30	100
Sokejima, 1998	52	100	31	40	100
Toker, 2012	58	100	46	40	100
Toppinen, 2009	54	100	54	20	100
Tsou, 2020	71	100	62	60	100

Newcastle-Ottawa Scale (NOS)
for cross-sectional studies

Selection: (Maximum 5 stars)
1) Representativeness of the sample:
a) Truly representative of the average in the target population. * (all subjects or random sampling)
b) Somewhat representative of the average in the target population. * (nonrandom sampling)
c) Selected group of users. d) No description of the sampling strategy.

2) Sample size: a) Justified and satisfactory. *
b) Not justified.

3) Non-respondents:
a) Comparability between respondents and non-respondents characteristics is established, and the response rate is satisfactory. *
b) The response rate is unsatisfactory, or the comparability between respondents and non-respondents is unsatisfactory.
c) No description of the response rate or the characteristics of the responders and the non-responders.

4) Ascertainment of the exposure (risk factor):
a) Validated measurement tool. **
b) Non-validated measurement tool, but the tool is available or described.*
c) No description of the measurement tool.

Comparability: (Maximum 2 stars)
1) The subjects in different outcome groups are comparable, based on the study design or analysis. Confounding factors are controlled.
a) The study controls for the most important factor (select one). *
b) The study control for any additional factor. *

Outcome: (Maximum 3 stars)
1) Assessment of the outcome:
a) Independent blind assessment. **
b) Record linkage. **
c) Self report. *
d) No description.

2) Statistical test:
a) The statistical test used to analyze the data is clearly described and appropriate, and the measurement of the association is presented, including confidence intervals and the probability level (p value). *
b) The statistical test is not appropriate, not described or incomplet.

Newcastle-Ottawa Scale (NOS)
for cohort studies

Selection (Maximum 4 stars)
1) Representativeness of the exposed cohort
a) truly representative of the average _______________ (describe) in the community *
b) somewhat representative of the average ______________ in the community*
c) selected group of users eg nurses, volunteers
d) no description of the derivation of the cohort

2) Selection of the non exposed cohort
a) drawn from the same community as the exposed cohort *
b) drawn from a different source
c) no description of the derivation of the non exposed cohort

3) Ascertainment of exposure
a) secure record (eg surgical records) *
b) structured interview *
c) written self report
d) no description

4) Demonstration that outcome of interest was not present at start of study
a) yes *
b) no

Comparability (Maximum 2 stars)
1) Comparability of cohorts on the basis of the design or analysis
a) study controls for _____________ (select the most important factor) *
b) study controls for any additional factor * (This criteria could be modified to indicate specific control for a second important factor.)

Outcome (Maximum 3 stars)
1) Assessment of outcome
a) independent blind assessment *
b) record linkage *
c) self report
d) no description

2) Was follow-up long enough for outcomes to occur
a) yes (select an adequate follow up period for outcome of interest) *
b) no

3) Adequacy of follow up of cohorts
a) complete follow up - all subjects accounted for *
b) subjects lost to follow up unlikely to introduce bias - small number lost - > ____ % (select an adequate %) follow up, or description provided of those lost) *
c) follow up rate < ____% (select an adequate %) and no description of those lost
d) no statement

Strengthening the Reporting of Observational studies in Epidemiology (STROBE) for cross-sectional and cohort studies

Section/Topic	Item #	Recommendation
**Title and abstract**	1	(*a*) Indicate the study's design with a commonly used term in the title or the abstract
		(*b*) Provide in the abstract an informative and balanced summary of what was done and what was found
**Introduction**		
Background/rationale	2	Explain the scientific background and rationale for the investigation being reported
Objectives	3	State specific objectives, including any prespecified hypotheses
**Methods**		
Study design	4	Present key elements of study design early in the paper
Setting	5	Describe the setting, locations, and relevant dates, including periods of recruitment, exposure, follow-up, and data collection
Participants	6	Give the eligibility criteria, and the sources and methods of selection of participants
Variables	7	Clearly define all outcomes, exposures, predictors, potential confounders, and effect modifiers. Give diagnostic criteria, if applicable
Data sources/ measurement	8*	For each variable of interest, give sources of data and details of methods of assessment (measurement). Describe comparability of assessment methods if there is more than one group
Bias	9	Describe any efforts to address potential sources of bias
Study size	10	Explain how the study size was arrived at
Quantitative variables	11	Explain how quantitative variables were handled in the analyses. If applicable, describe which groupings were chosen and why
Statistical methods	12	(*a*) Describe all statistical methods, including those used to control for confounding
		(*b*) Describe any methods used to examine subgroups and interactions
		(*c*) Explain how missing data were addressed
		(*d*) If applicable, describe analytical methods taking account of sampling strategy
		(*e*) Describe any sensitivity analyses
**Results**		
Participants	13*	(a) Report numbers of individuals at each stage of study—eg numbers potentially eligible, examined for eligibility, confirmed eligible, included in the study, completing follow-up, and analysed
		(b) Give reasons for non-participation at each stage
		(c) Consider use of a flow diagram
Descriptive data	14*	(a) Give characteristics of study participants (eg demographic, clinical, social) and information on exposures and potential confounders
		(b) Indicate number of participants with missing data for each variable of interest
Outcome data	15*	Report numbers of outcome events or summary measures
Main results	16	(*a*) Give unadjusted estimates and, if applicable, confounder-adjusted estimates and their precision (eg, 95% confidence interval). Make clear which confounders were adjusted for and why they were included
		(*b*) Report category boundaries when continuous variables were categorized
		(*c*) If relevant, consider translating estimates of relative risk into absolute risk for a meaningful time period
Other analyses	17	Report other analyses done—eg analyses of subgroups and interactions, and sensitivity analyses
**Discussion**		
Key results	18	Summarise key results with reference to study objectives
Limitations	19	Discuss limitations of the study, taking into account sources of potential bias or imprecision. Discuss both direction and magnitude of any potential bias
Interpretation	20	Give a cautious overall interpretation of results considering objectives, limitations, multiplicity of analyses, results from similar studies, and other relevant evidence
Generalisability	21	Discuss the generalisability (external validity) of the study results
**Other information**		
Funding	22	Give the source of funding and the role of the funders for the present study and, if applicable, for the original study on which the present article is based

*Give information separately for cases and controls in case-control studies and, if applicable, for exposed and unexposed groups in cohort and cross-sectional studies.

Note: An Explanation and Elaboration article discusses each checklist item and gives methodological background and published examples of transparent reporting. The STROBE checklist is best used in conjunction with this article (freely available on the Web sites of PLoS Medicine at http://www.plosmedicine.org/, Annals of Internal Medicine at http://www.annals.org/, and Epidemiology at http://www.epidem.com/). Information on the STROBE Initiative is available at at www.strobe-statement

Appendix 3. Meta-analysis using the most adjusted and crude risk of cardiovascular disease after a burnout.

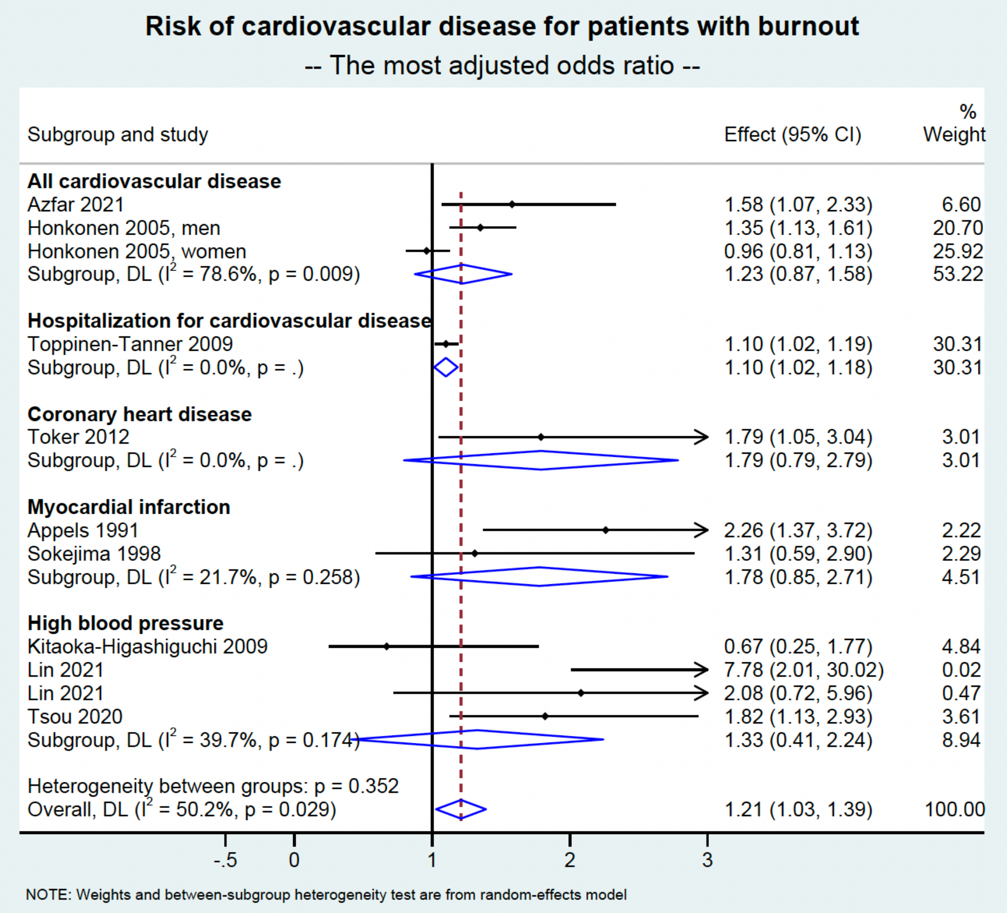

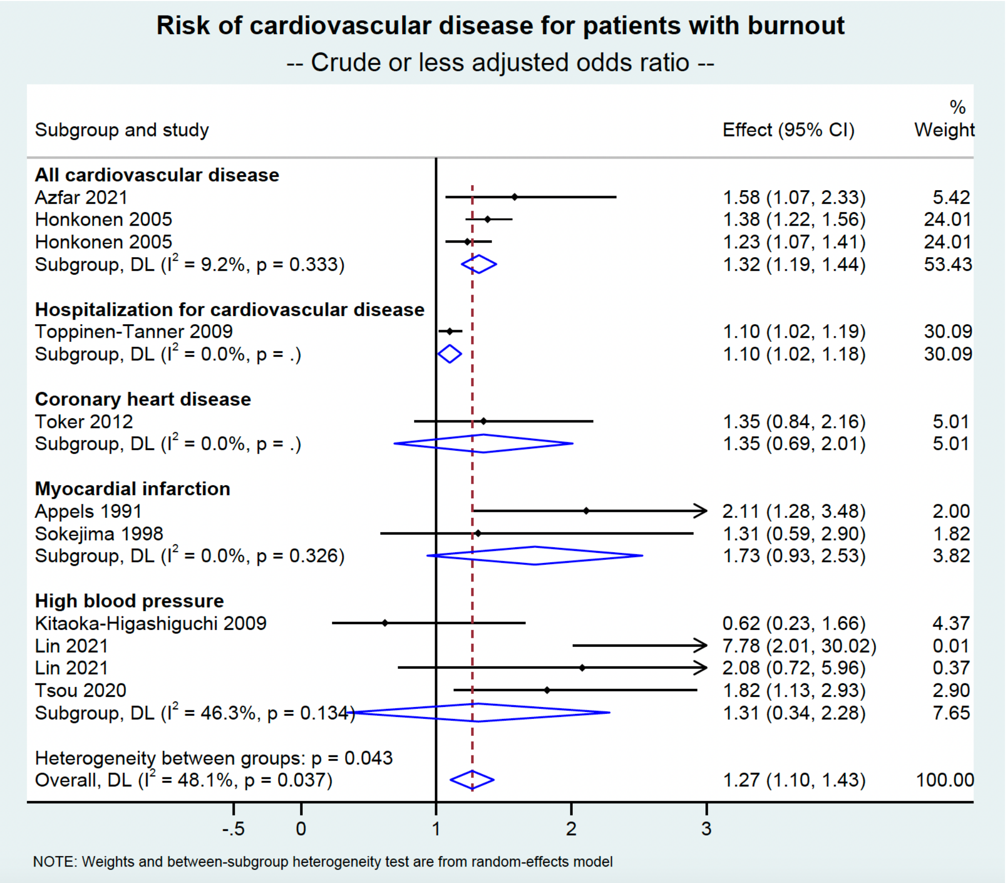

Appendix 4. Metafunnels, based on data from the most adjusted and crude risk of cardiovascular disease after a burnout.

Each dot represents a single study, with its corresponding risk (x axis) and its associated standard error of the risk (y-axis). Large high-powered studies are placed towards the top, and smaller low-powered studies towards the bottom. The plot should ideally resemble a pyramid or inverted funnel, with scatter due to sampling variation. Studies outside funnel plot are likely to present bias (94).

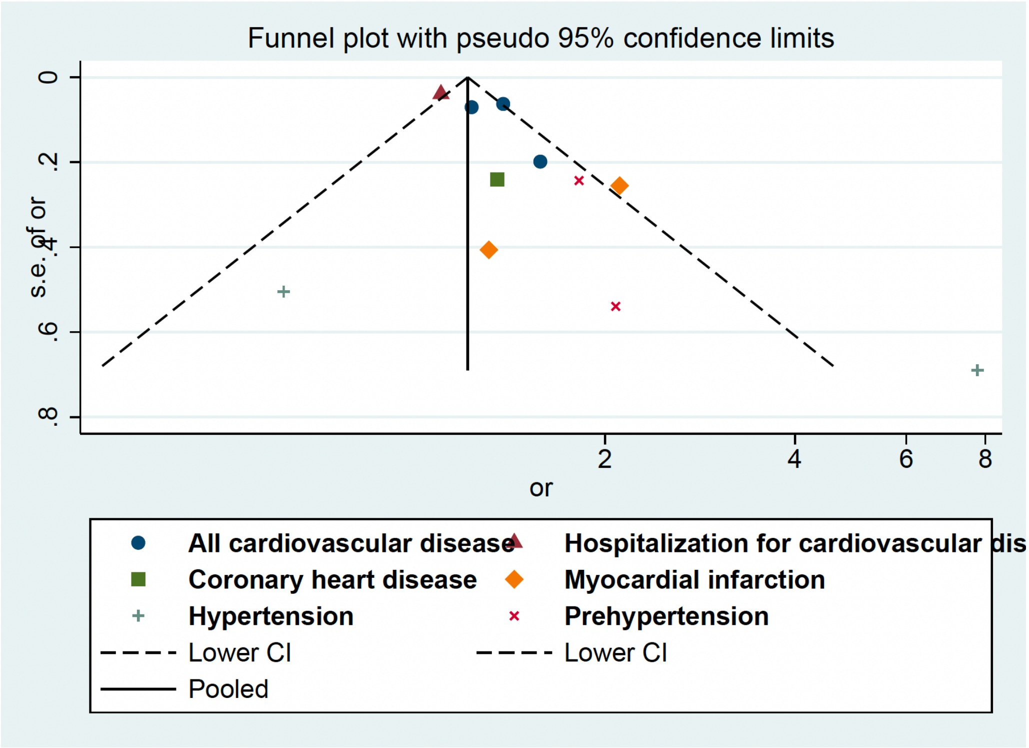

Supplement: Appendix 1 — Details for the search strategy used within each database. [file Presentation_1.pdf]
